# Supplementary material for: Splice-Junction-Based Mapping of Alternative Isoforms in the Human Proteome
Source: Cell Rep. Author manuscript; Available in PMC 2020 Jan 15. (PMC6961840; doi:10.1016/j.celrep.2019.11.026)
Supplement: 2 [file NIHMS1546469-supplement-2.zip › DF1/PXD006675/AtrialSeptum/AtrialSeptum_30_TTN_INVLEPPK.pdf]

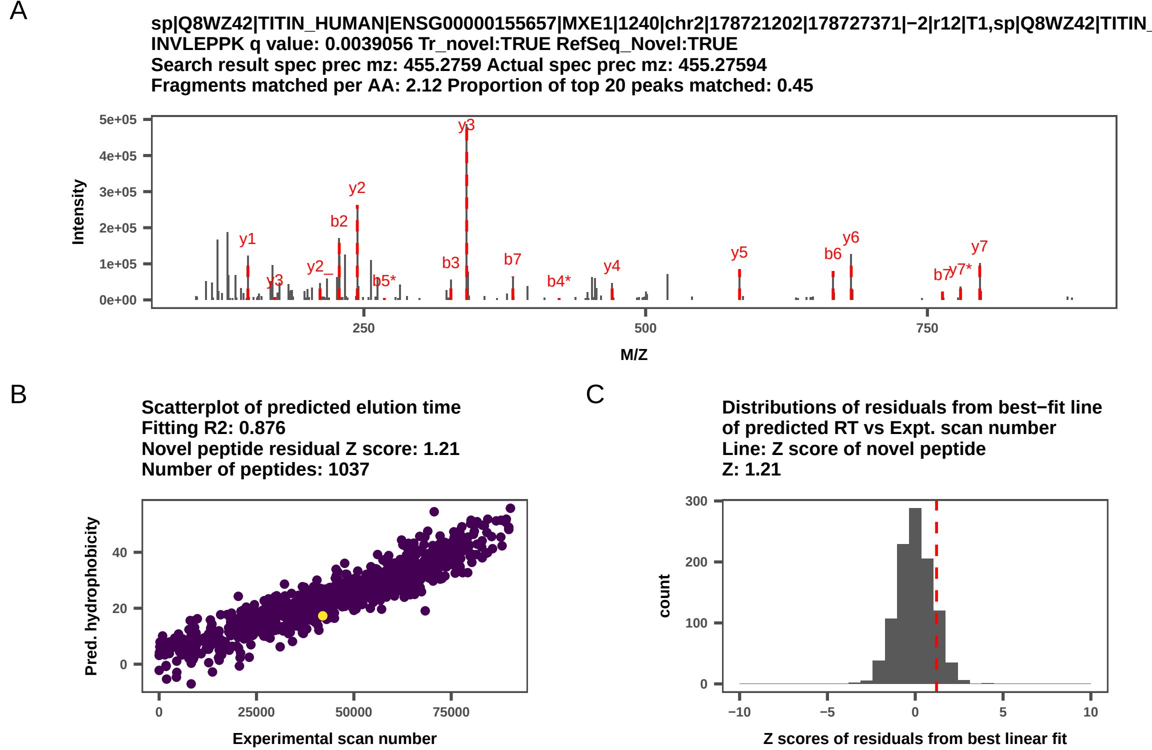
A
sp|Q8WZ42|TITIN_HUMAN|ENSG00000155657|MXE1|1240|chr2|178721202|178727371|−2|r12|T1,sp|Q8WZ42|TITIN_
INVLEPPK q value: 0.0039056 Tr_novel:TRUE RefSeq_Novel:TRUE
Search result spec prec mz: 455.2759 Actual spec prec mz: 455.27594
Fragments matched per AA: 2.12 Proportion of top 20 peaks matched: 0.45
Intensity
5e+05
4e+05
3e+05
2e+05
1e+05
0e+00
y1
y3
y2_
b2
y2
b5*
b3
y3
b7
b4*
y4
y5
b6
y6
b7
y7*
y7
250
500
750
M/Z
B
Scatterplot of predicted elution time
Fitting R2: 0.876
Novel peptide residual Z score: 1.21
Number of peptides: 1037
Pred. hydrophobicity
40
20
0
0
25000
50000
75000
Experimental scan number
C
Distributions of residuals from best−fit line
of predicted RT vs Expt. scan number
Line: Z score of novel peptide
Z: 1.21
count
300
200
100
0
−10
−5
0
5
10
Z scores of residuals from best linear fit
